# The Use of Placebo and Non-Specific Therapies and Their Relation to Basic Professional Attitudes and the Use of Complementary Therapies among German Physicians – A Cross-Sectional Survey

**DOI:** 10.1371/journal.pone.0092938

**Published:** 2014-04-02

**Authors:** Klaus Linde, Clara Friedrichs, Anna Alscher, Stefan Wagenpfeil, Karin Meissner, Antonius Schneider

**Affiliations:** 1 Institute of General Practice, Technische Universität München, Munich, Germany,; 2 Institute of Medical Biometry, Epidemiology and Medical Informatics, Universitätsklinikum des Saarlandes, Homburg, Germany,; 3 Institute of Medical Psychology, Ludwig-Maximilians-University, Munich, Germany; University of Louisville, United States of America

## Abstract

We aimed to investigate the use of placebos (e.g. saline injections) and non-specific treatments (e.g. vitamin supplements in individuals without a relevant deficiency) among physicians working in private practices in Germany, and how such use is associated with the belief in and the use of complementary and alternative treatments, and basic professional attitudes. A four-page questionnaire was sent to nationwide random samples of general practitioners (GP), internists and orthopaedists working in private practices. The response rate was 46% (935 of 2018). 24% of GPs, 44% of internists and 57% of orthopaedists had neither used pure placebos nor non-specific therapies in the previous 12 months. 11% percent of GPs, 12% of internists and 7% of orthopaedists had exclusively used pure placebos; 30%, 33% and 26%, respectively, had exclusively used non-specific therapies; 35%, 12% and 9% had used both. Age, sex and agreement to the statement that physicians should harness placebo effects were not significantly associated with any pattern of use. Exclusive use of pure placebos was associated with being a GP, being an internist, and having unorthodox professional views. In addition to these three factors, a lower use of CAM therapies and a wish for having more time was associated with the exclusive use of non-specific therapies. Among physicians using both pure placebo and non-specific therapies, heterodox views were also somewhat more pronounced. However, associations were particularly strong for being a GP (Odds ratio 11.6 (95%CI 6.41; 21.3)) and having orthodox views (Odds ratio 0.10 (95%CI 0.06; 0.18)) among this group. In conclusion, the use of placebos and non-specific treatments varies strongly between medical specialties and is associated with basic professional attitudes. The findings support the view that the use of placebos and, in particular, of non-specific therapies is primarily a coping behaviour for difficult and uncertain situations.

## Introduction

There is clear evidence that many physicians in primary and secondary care, to some extent, use treatments for patients or in situations even though they hold a personal view that these treatments only have non-specific or placebo effects. This evidence mainly comes from two quite different lines of research. The first line consists of quantitative surveys on the use of placebos outside of clinical trials [1]–[4]. For example, a recent survey among UK GPs found that 12% had used treatments that do not contain any active components such as saline injections or sugar pills (called placebos or ‘pure’ placebos) at least once in their professional life. 97% had used treatments containing active or potentially active components that did not have a specific effect on the condition treated, such as antibiotics for viral infections or vitamin supplements in individuals without relevant deficiency (called ‘impure’ placebos or non-specific treatments) [4]. While other motivations are also usually examined, an implicit assumption in most of these surveys is that eliciting placebo effects is a major reason for providing such treatments. For example, one major survey defined placebo treatment to participants as “a treatment whose benefits derive from positive patients expectations” [3].

The second line of research is mostly qualitative research on difficult or inappropriate prescribing decisions (e.g. [5]–[9]). While the word placebo rarely ever shows up, these studies deal with the motivations for using interventions that, in placebo surveys, are considered typical examples of non-specific treatments or ‘impure’ placebos (e.g. antibiotics for viral infections). The findings of this research suggest that uncertainty, perceived patients' expectations and pressure to act are the main reasons to use such treatments. During their training, physicians internalize a professional ideal that any treatment should have specific activity and should be administered or prescribed only when necessary [5]. However, this ideal conflicts with experiences in everyday practice where physicians face considerable uncertainty. From this perspective, the use of non-specific treatments could be seen as a problem-solving tool that can be used to manage a variety of difficult situations in routine practice [9].

Interpreting the use of non-specific treatments (and to some extent also of placebos) as a somewhat ambiguous strategy to deal with difficult and uncertain situations is in line with empirical findings. In placebo surveys, many physicians report patient expectations and avoiding conflicts as motivations for using placebos and non-specific treatments in addition to eliciting placebo effects [1]–[4], [10]. In ambulatory care, non-specific treatments are used much more often than pure placebos [1]–[4]. Applying a placebo implies a very conscious decision and, in most cases, giving deceptive information. Applying non-specific treatments is usually a less challenging option. There is also some data suggesting that the use of non-specific treatments is particularly widespread among GPs [1], [2], [4], [10], [11]. GPs see many patients for whom it is difficult to make a clear diagnosis [12] and who have minor ailments [13]. Non-specific treatments should be a less relevant option for specialists, who mainly see pre-selected cases with more advanced disease often receiving multiple treatments. One could also expect an association between the use of complementary and alternative medicine (CAM) and the use of non-specific treatments [14]. To those who believe in a given CAM modality, it offers - or seems to offer - ‘specific’ diagnostic and therapeutic solutions for patients for whom conventional medicine does not provide satisfactory solutions. As a result, convinced CAM users might report less use of treatments they consider non-specific. Finally, one would expect that basic professional attitudes have a relevant role on the behaviour of physicians. For example, a physician firmly believing that medicine has to be based on sound science could be less likely to use placebos, non-specific treatments and CAM treatments.

In the survey presented in this paper we aimed to investigate the use of placebos and non-specific treatments among GPs, internists and orthopaedists working in private practice in Germany. We also asked for beliefs in and the use of major CAM modalities and basic professional attitudes to investigate how these issues are related to the use of placebos and non-specific treatments.

## Methods

### Design

The study was a postal, cross-sectional survey. It was approved by the ethical review board of the Medical Faculty of the Technische Universität München. We randomly (using the random sampling function in SPSS) selected 700 GPs, 700 internists and 700 orthopaedists working in private practice from a commercially available database (www.adressendiscount.de) with the addresses and accredited specializations of more than 90% of all physicians providing ambulatory care in Germany. In late October 2012 selected physicians received a letter with information on the study, a four-page questionnaire and a pre-stamped envelope. Non-responders received up to two reminders until early December 2012. Data was entered into an SPSS database which was closed on February 28, 2013.

### Questionnaire

The rationale, the development and the details of the questionnaire have been reported elsewhere [14]. Briefly, the questionnaire consisted of 50 items divided into five blocks. Block A consisted of four questions on the use of placebos (use, frequency of use in the last 12 months, repeated use in single patients and availability of placebo preparations in the practice) and block B of three questions on the use of non-specific treatment (use, frequency of use in the last 12 months and types of interventions used). We used the term “non-specific treatment” because cognitive interviews during the development have shown that physicians had difficulties with the term “impure placebo” [14]. Block C included 21 questions on the belief in the specific effects and on the use of seven complementary therapies (acupuncture, homeopathy, chirotherapy, osteopathy, herbal medicine, other classical naturopathic treatments, vitamins/microelements (further treatments could be added)), and on the qualifications for these therapies. Block D consisted of 13 statements on basic professional attitudes. Physicians were asked to indicate the level of agreement on a four-point scale. Based on theoretical considerations [5], [14] and exploratory factor analysis, answers to 12 statements were summarized on three scales: orthodox views (five statements expressing conventional medico-scientific views, e.g. “whenever possible only evidence-based treatments should be used”); heterodox views (three statements related to the limitations of conventional medicine and usefulness of CAM, e.g. “in my daily practice I am confronted with many patients in which the classical knowledge from textbooks is insufficient”), and time/patient-doctor relationship (four items addressing the need of time and the relevance of the patient-doctor relationship). We also analyzed the statement on harnessing placebo effects (“as a physician one should intensively harness positive psychological effects (e.g. ‘drug physician’)”) separately because it was a central consideration. In block E socio-demographic and practice characteristics were documented.

### Statistics

All analyses were performed using IBM SPSS Statistics 21 using all available data. Data were analyzed descriptively for the three physician groups using absolute counts, percentages, means and standard deviations, medians and quartiles, as appropriate. 95% confidence intervals (95% CI) for frequencies were estimated using the bootstrapping function in SPSS. P-values for comparisons between the three specialties were calculated using Chi^2^-tests, a Kruskal-Wallis test, and analysis of variance. To investigate which physician characteristics were associated with the exclusive use of placebos, the exclusive use of non-specific therapies and the use of both placebos and non-specific therapies we performed multivariate multinomial regression (using neither use as reference category). Independent variables for the final model were age, sex, specialty (using orthopaedists as reference group), the four scales regarding basic professional attitudes, CAM use and CAM belief. Assuming a response rate of about 40%, we sent out 700 questionnaires to have 80% power to detect a 12% difference in the use of non-specific treatments between GPs (assumed prevalence 60%) and internists (48%) using a two-tailed p-value of 0.05 (n per group 288 patients, calculation with G*Power 3.1.2 for Fisher's exact test).

## Results

Of the 2100 questionnaires sent out, 72 could not be delivered (address no longer valid). Furthermore, six physicians were actually working in a hospital, three had retired and one had died. So our final sample consisted of 2018 physicians (685 GPs, 661 internists and 669 orthopaedists). 935 (46%; 319 GPs, 311 internists and 305 orthopaedists) sent back a completed questionnaire until the closing of the database. 41% of GPs, 20% of internists and 10% of orthopaedists were women (p-value for differences between specialties <0.001) and the mean age was 55 years among GPs, 53 among internists and 54 among orthopaedists (p = 0.19).

The use of placebos in the previous 12 months was reported more frequently by GPs (46%; 95%CI 40% to 51%) than by internists (24%; 95%CI 19% to 29%) and orthopaedists (17%; 95%CI 13% to 21%; see Table 1). Among those who had used a placebo, the median frequency of use was 2 per year (first and third quartile 1 and 5) with minor differences between specialties. 35 physicians (5%) reported that they had prefabricated placebos tablets or globules in practice. However, two physicians named products which actually were supplements.

**Table 1:** Use of pure placebos and non-specific therapies among respondents.

	GPs (n = 319)	Internists (n = 305)	Orthopaedists (n = 311)
**Use of placebos**			
Ever use	168 (52%; 43 to 54%)	92 (30%; 25 to 35%)	60 (19%; 15 to24%)
Use in the last 12 months			
- never	173 (54%; 49 to 60%)	233 (76%; 72 to 81%)	259 (83%; 79 to 88%)
- 1 to 5 times	111 (35%; 30 to 40%)	55 (18%; 24 to 23%)	30 (10%; 6 to 13%)
- 6 to 20 times	24 (8%; 4 to 10%)	14 (5%; 2 to 7%)	13 (4%; 2 to 7%)
- 21 to 50 times	8 (3%; 1 to 4%)	1 (<1%; 0 to 1%)	3 (1%; 0 to 2%)
- more than 50 times	3 (1%; 0 to 2%)	2 (1%; 0 to 2%)	6 (2%; 1 to 4%)
- Median frequency (first/third quartile) among users	2 (1/5)	2 (1/5)	5 (2/10)
Use in single patients more than once	79 (25%; 20 to 30%)	42 (14%; 10 to 18%))	28 (9%; 6 to 12%))
Prefabricated placebo in practice	18 (6%; 4 to 9%)	14 (5%; 2 to 7%)	3 (1%; 0 to 2%)
**Use of non-specific therapies**			
Ever use	213 (67%; 62 to 72)	143 (47%; 28 to 38%)	119 (38%; 33 to 44%)
Use in the last 12 months			
- never	111 (35%; 30 to 40%)	168 (55%; 50 to 61%)	200 (64%; 59 to 69%)
- 1 to 5 times	37 (12%; 8 to 15%)	26 (9%; 6 to 12%)	22 (7%; 5 to 10%)
- 6 to 20 times	99 (31%; 26 to 36%)	64 (21%; 16 to 26%)	45 (15%; 11 to 19%)
- 21 to 50 times	39 (12%; 9 to 16%)	28 (9%; 6 to 13%)	12 (4%; 2 to 6%)
- more than 50 times	33 (10%; 7 to 14%)	19 (6%; 4 to 9%)	32 (10%; 7 to 14%)
- Median frequency (first/third quartile) among users	15 (10/48)	20 (10/50)	20 (10/100)
Types of treatments used			
- vitamins	136 (43%; 38 to 48%)	81 (27%; 22 to 32%)	71 (23%; 18 to 28%)
- herbal remedies	135 (42%; 37 to 47%)	91 (30%; 25 to 35%)	55 (18%; 14 to 22%)
- minerals and micro elements	112 (35%; 30 to 41%)	80 (26%; 21 to 31%)	47 (15%; 11 to 19%)
- homeopathic remedies	106 (33%; 28 to 39%)	47 (15%; 11 to 20%)	62 (20%; 15 to 24%)
- antibiotics	108 (34%; 29 to 39%)	51 (17%; 13 to 21%)	3 (1%; 0 to 2%)
- analgesics	44 (14%; 10 to 18%)	17 (6%; 3 to 8%)	14 (5%; 2 to 7%)
**Pattern of use in the last 12 months**			
- neither	76 (24%; 19 to 29%)	133 (44%; 38 to 50%)	177 (57%; 51 to 62%)
- pure placebos only	35 (11%; 8 to 15%)	35 (12%; 8 to 15%)	23 (7%; 5 to 10%)
- non-specific therapies only	97 (30%; 25 to 35%)	100 (33%; 27 to 38%)	82 (26%; 21 to 31%)
- both	111 (35%; 30 to 40%)	37 (12%; 9 to 16%)	29 (9%; 6 to 13%)

Numbers are absolute frequencies (percentages; 95%-CI) unless otherwise indicated.

Non-specific treatments were used in the last 12 months by 65% (95%CI 60% to 70%) of GPs, 45% (95%CI 39% to 51%) of internists and 36% (95%CI 30% to 41%) of orthopaedists. The median frequency of use among users was 20 (first and third quartile 10 and 50; no statistically significant differences between groups). Vitamins, herbal remedies, minerals and microelements and homeopathic remedies were most often used as non-specific treatments. Antibiotics were used by 34% (95%CI 29% to 39%) of GPs, 17% (95%CI 13% to 21%) of internists and only 1% (95%CI 0% to 2%) of orthopaedists.

Overall, 24% (95%CI 19% to 29%) of GPs, 44% (95%CI 38% to 50%) of internists and 57% (95%CI 51% to 62%) of orthopaedists had neither used pure placebos nor non-specific therapies in the previous 12 months. 11% (95%CI 8% to 15%) of GPs, 12% (95%CI 8% to 15%) of internists and 7% (95%CI 5% to 10%) of orthopaedists reported the exclusive use of pure placebos. 30% (95%CI 25% to 35%) of GPs, 33% (295%CI 7% to 38%) of internists and 26% (95%CI 21% to 31%) had exclusively used non-specific therapies. The use of both placebos and non-specific therapies was reported by 35% (95%CI 30 to 40%) of GPs, 12% (95%CI 9% to 16%) of internists and 9% (95%CI 6% to 13%) of orthopaedists.

On average, internists agreed more with orthodox views and less with heterodox views than the two other groups (see Table 2). GPs agreed with heterodox views more than orthopaedists and clearly more than internists. GPs also agreed more with statements on the need of more time and the patient-doctor relationship and they were more positive about harnessing placebo effects than internists and orthopaedists, though the differences were relatively small. GPs and orthopaedists considered CAM treatments to be specifically active more often than internists but there was strong variation within all three physician groups. The use of CAM therapies was particularly frequent among orthopaedists and least prevalent among internists.

**Table 2:** Basic professional attitudes, belief in and use of CAM therapies.

	GPs	Internists	Orthopaedists
**Basic professional attitudes (1 = strong agreement, 4 = strong disagreement; n = 897)**			
Scale orthodox views (5 items)	2.05 (1.99; 2.11)	1.80 (1.75; 1.86)	2.06 (2.01; 2.12)
Scale heterodox views (3 items)	2.37 (2.30; 2.44)	2.89 (2.81; 2.95)	2.56 (2.50; 2.62)
Scale time/patient-doctor relationship (4 items)	1.57 (1.52; 1.63)	1.71 (1.65; 1.77)	1.67 (1.61; 1.73)
Single item harnessing placebo effects	1.85 (1.75; 1.94)	1.96 (1.87; 2.05)	1.98 (1.89; 2.07)
**Average CAM belief (1 = only placebo, 5 = specifically active; n = 873)**			
Summary scale 7 therapies	3.49 (3.41; 3.58)	3.08 (2.99; 3.17)	3.51 (3.44; 3.58)
**Number of CAM therapies used more often than once per week (n = 874)**			
- none	46 (15%; 11 to 19%))	148 (52%; 45 to 57%)	11 (4%; 2 to 6%)
- one	51 (17%; 13 to 21%)	80 (28%; 23 to 33%)	31 (11%; 8 to 15%)
- two	82 (27%; 23 to 33%)	28 (10%; 7 to 13%)	93 (33%; 28 to 39%)
- three to four	90 (19%; 24 to 35%))	26 (9%; 6 to 13%)	95 (34%; 29 to 39%)
- five to seven	35 (11%; 8 to 15%)	5 (2%; 0 to 4%)	50 (18%; 14 to 22%)

Values are means (95%-CI) or absolute frequencies (percentages; 95%CI).

Table 3 summarizes the findings from multivariate multinomial regression analyses on associations between physician characteristics and different patterns of use of placebos and non-specific therapies. In general, age, sex and agreement to the statement that physicians should harness placebo effects were not significantly associated with any pattern of use. Exclusive use of pure placebos was associated with being a GP, an internist, and more disagreement with orthodox views. In addition to these three factors, a lower belief in specific effects of CAM therapies and a higher value on the time and relationship scale were associated with exclusive use of non-specific therapies. The same five factors were associated with using both pure placebos and non-specific therapies, but associations were particularly strong for being a GP (OR 11.6 (95%CI 6.26; 21.6)) and agreeing with orthodox views (OR  = 0.11 (95%CI 0.06; 0.19)) among this group.

**Table 3:** Factors associated with the pattern of the use of placebos and non-specific treatments in multivariate multinomial regression (n = 796, r^2^ = 0.30 (Nagelkerke)).

	Only pure placebos OR (95%CI)	Only non-specific therapies OR (95%CI)	Both OR (95%CI)
Age (per year)	1.00 (0.97; 1.04)	1.00 (0.98; 1.03)	1.01 (0.98; 1.04)
Sex female	1.13 (0.60; 2.11)	1.17 (0.74; 1.87)	0.69 (0.39; 1.22)
GP	3.41 (1.66; 7.04)	3.46 (2.07; 5.76)	11.8 (6.35; 22.1)
Internist	3.41 (1.60; 7.26)	2.47 (1.45; 4.21)	3.57 (1.77; 7.21)
Orthodox views	0.38 (0.20; 0.72)	0.21 (0.13; 0.33)	0.11 (0.07; 0.20)
Heretic views	0.93 (0.55; 1.57)	1.24 (0.85; 1.79)	1.44 (0.92; 2.25)
Time & relationship	1.19 (0.70; 2.01)	2.01 (1.37; 2.95)	2.07 (1.29; 3.33)
Harnessing placebo effects	1.22 (0.88; 1.69)	1.24 (0.98; 1.56)	1.32 (0.98; 1.77)
CAM belief	1.16 (0.73; 1.83)	0.42 (0.30; 0.58)	0.47 (0.32; 0.70)
CAM use	1.15 (0.87; 1.53)	1.12 (0.92; 1.38)	1.14 (0.89; 1.44)

Odds ratios (OR) >1 indicate more frequent use compared to physicians using neither placebos nor non-specific treatments.

## Discussion

Physicians working in private practice in Germany use non-specific treatments much more frequently than (pure) placebos. The use of both placebos and non-specific therapies is more widespread among GPs than among internists and - contrary to our initial expectations [14] - lowest among orthopaedists. Professional attitudes and the belief in and the use of CAM treatments differ markedly within and between medical specialties with internists being, on average, most sceptical and orthopaedists using CAM treatments most often. In multivariate regression analyses, the factors most strongly associated with the use of placebos and non-specific treatments were being a GP (higher likelihood of use) and holding orthodox views (lower likelihood). Stronger belief in specific effects of CAM but not more frequent use of CAM was associated with a lower use of non-specific treatments.

Our results confirm previous findings from other countries that non-specific treatments are used much more frequently than placebos [1], [3], [4], [10], [11], [15], [16]. Compared to physicians working in ambulatory care in other countries, the proportion of German physicians using placebos seems relatively high while the reported use of non-specific treatments does not seem unusual. While our prevalence estimates of the use of placebos and non-specific treatment are of some interest, the clear strengths and novel aspects of our study are regarding the investigation of differences between physician specialties and of the influence of professional attitudes and CAM belief or use. Indirect comparison of different surveys already suggested that GPs use placebos and non-specific treatments more often than specialists [1]. But so far only one Danish survey of 503 physicians directly compared GPs, private specialists and hospital-based physicians [10]. 86% of general practitioners had used at least one of such treatments in the last year compared to 54% of hospital doctors and 41% of private specialists. Our results show that there are also significant differences between different types of private specialists. The differences between GPs and internists were expected and seem plausible (see introduction), but the findings on orthopaedists surprised us. To the best of our knowledge, orthopaedists in private practice have not been included in previous placebo surveys [1]. We had included this group because it seemed of considerable interest. Orthopaedists are mainly trained in hospitals where they typically treat selected patients with surgical procedures. If orthopaedists later provide ambulatory care in private practice they very often see patients suffering from chronic pain or functional musculoskeletal disorders for which their training should have provided only few specific solutions. As we expected, orthopaedists agreed less than GPs and internists to the statement that their post-graduate medical training prepared them well for their work in private practice. Nevertheless, orthopaedists reported the lowest use of placebos and non-specific treatments and instead use and believe in CAM modalities – in particular, chirotherapy, acupuncture and osteopathy (detailed results will be reported elsewhere) – much more often than GPs and internists. Our finding that belief in the efficacy of CAM modalities is associated with lower reporting of the use of non-specific treatments *and* no association with actual CAM use would fit with our assumption that whether a treatment is considered specific or non-specific depends on the view of the individual physician. The differences between specialities regarding basic professional attitudes might be due to a considerable extent to personal traits already influencing the choice of work field. However, these differences probably at least partly also reflect experiences in real world practice.

About a quarter of GPs and about half of internists and orthopaedists claimed to manage their daily routine without any use of placebos or non-specific treatments. Furthermore, the agreement to the statement that “as a physician one should intensively harness positive psychological effects (e.g. ‘drug physician’)” did not show a strong association with placebo or non-specific treatment use. This could indicate that many physicians think that placebos or non-specific treatments are not necessarily needed for harnessing placebo effects. This leads to the central question about whether using placebos and non-specific treatments is ethically and professionally acceptable [17]. In a report on placebo use, the American Medical Association Council on Ethical and Judicial Affairs stated that the use of placebos or non-specific therapies is not ethically acceptable if it serves “the convenience of the physician rather than to promote the well-being of the patient” [18]. Two basic scenarios of “convenience” prescriptions have been distinguished: one where the physician tries to get the patient to stop complaining without providing adequate information on the treatment, and a second where the physicians states that he considers the treatment demanded by the patient unnecessary but finally prescribes it [19]. While we think that many convenience prescriptions can and should be avoided, the simple dichotomy between convenience and benevolent prescription does not reflect the complexity of the patient-physician relationship in the real world [9], [20]. There are multiple situations in medical practice in which it is difficult to draw a clear line of what is an unacceptable behaviour and what justifiable human care.

When interpreting the findings of our survey, several limitations have to be kept in mind. Less than half of the physicians contacted filled in the questionnaire and it is not clear whether the prevalence estimates found among respondents can be generalized. However, a main focus of our survey was the comparison between medical specialties. Given the very similar response rates in the three groups it seems unlikely that reasons for selection differ between the groups. Therefore, the marked differences between medical specialities are very likely to be a real phenomenon. Due to the unclear and subjective distinction between specific and non-specific treatments [1], [21], any prevalence estimates on non-specific treatment use have to be interpreted with great caution. For example, the CAM treatment considered specific by a believer might be reported as non-specific treatment by a sceptic. The very frequent use of non-specific treatments among GPs reported in the UK [4] might simply reflect that physicians in this country are more aware of the limited evidence many interventions have. At the same time, one can speculate that there is under-reporting of non-specific treatment use due to social desirability. Our “measurement” of basic professional attitudes can be considered only a first attempt and should not be considered a properly validated instrument. The cognitive interviews performed during the development phase made clear that the issues addressed are very difficult to grasp quantitatively in a standardized questionnaire [14]. Finally, while our analyses were based on pre-specified assumptions they have to be considered exploratory.

In conclusion, we think that our findings support the view that the use of placebos and, in particular, of non-specific therapies is a coping behaviour for difficult and uncertain situations. Furthermore, in many situations the hope to elicit placebo effects is probably more a justification than a primary motivation to use such treatment. Qualitative studies are needed to better understand why and in which situations physicians use placebos or non-specific treatments. Quantitative studies could investigate, for example, to what extent personality traits, styles of practicing (e.g. paternalistic vs. cooperative) or strategies to deal with uncertainty influence such use. A priority for future research should be to investigate how physicians not using placebos and non-specific treatment manage difficult situations. Based on the findings of such research it should be possible to develop educational strategies to reduce the inappropriate use of placebos and non-specific treatments. We doubt that it is possible or adequate to completely eliminate the use of placebos and non-specific treatment from clinical practice. In this respect, research on how such interventions can be applied in an ethically acceptable manner is clearly desirable.
